# FGL2-induced metabolic dysregulation in enteric neural crest cells provides insight into Hirschsprung disease pathogenesis

**DOI:** 10.1016/j.isci.2025.113423

**Published:** 2025-08-22

**Authors:** Jichang Han, Xiaoyang Liu, Yixuan Wang, Qiongqian Xu, Dong Sun, Xintao Zhang, Xixi He, Chuncan Ma, Xue Ren, Jian Wang, Yaru Mou, Qiangye Zhang, Dongming Wang, Weijing Mu, Peimin Hou, Aiwu Li

**Affiliations:** 1 Department of Pediatric Surgery, Qilu Hospital of Shandong University, Jinan, China; 2 The First Affiliated Hospital of Jishou University, Xiangxi Autonomous Prefecture, China; 3 Department of Cardiology, Shandong Provincial Hospital Affiliated to Shandong First Medical University, Jinan, Shandong, China

**Keywords:** Gastroenterology, Human metabolism, Molecular mechanism of gene regulation

## Abstract

Hirschsprung disease (HSCR) is a congenital disorder characterized by the absence of enteric neural crest cells (ENCCs) in aganglionic segments of the intestine. Here, we report that fibrinogen-like protein 2 (FGL2) is upregulated in aganglionic colon tissues from patients with HSCR. *In vitro* experiments using primary ENCCs isolated from embryonic mouse colon showed that FGL2 stimulation increased ENCC apoptosis. Mechanistically, FGL2 enhances oxidative phosphorylation (OXPHOS) via the activation of the JAK2–STAT3 signaling pathway, leading to reactive oxygen species (ROS) accumulation and subsequent apoptosis. Overall, these findings identify FGL2 as a potential pathogenic factor in HSCR that impacts ENCC survival and provide mechanistic insight into the metabolic regulation of ENCCs in the context of HSCR.

## Highlights

• FGL2 expression is upregulated in aganglionic segments of patients with HSCR
• FGL2 promotes apoptosis in enteric neural crest cells (ENCCs) *in vitro*
• FGL2 induces OXPHOS elevation and ROS overproduction in ENCCs
• JAK2–STAT3 signaling mediates FGL2-driven metabolic reprogramming

## Graphical abstract

**Figure:**
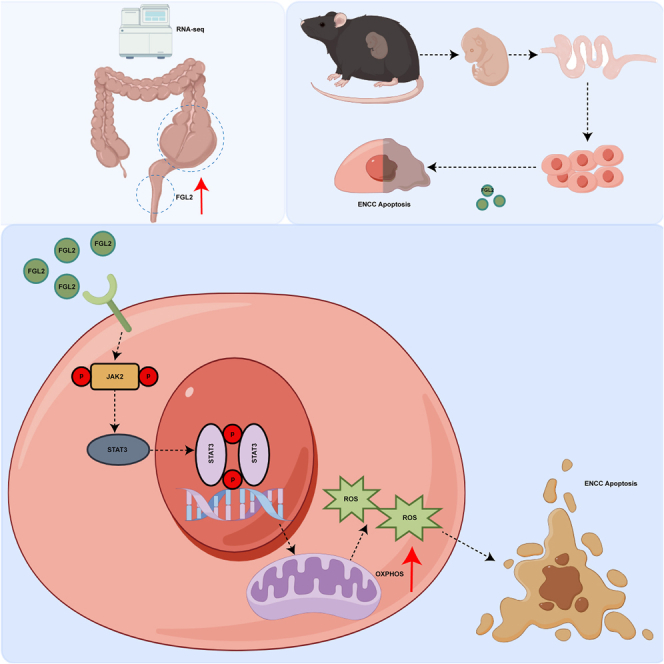

## Introduction

Hirschsprung disease (HSCR) is the most common congenital gastrointestinal motility disorder, affecting approximately 1 in 5,000 live births.^1^ It is characterized by the absence of enteric neural crest cells (ENCCs) in the distal gut, resulting in varying lengths of aganglionic segments and intestinal obstruction.^2^ This pathology arises from defects in ENCC growth, migration, and/or differentiation during embryonic development. While cumulative genetic variants with minor individual effects are considered the primary drivers of HSCR, these variants likely converge on shared pathogenic pathways that ultimately lead to the disease.^2^

Recent studies have shed light on the genetic and molecular mechanisms underlying HSCR, particularly focusing on ENCC functions such as migration, proliferation, differentiation, and apoptosis.^1^^,^^3^ However, the pathogenesis remains incompletely understood, especially concerning the role of metabolic regulation in ENCC survival and differentiation. Metabolic processes, particularly oxidative phosphorylation (OXPHOS), play a vital role in cellular homeostasis, and perturbations in OXPHOS have been associated with impaired ENCC differentiation and survival in HSCR. Perturbed OXPHOS levels have been strongly associated with poor survival and impaired the differentiation of ENCCs in HSCR.^2^^,^^4^

Fibrinogen-like protein 2 (FGL2), a secreted glycoprotein belonging to the fibrinogen-related protein family, has been implicated in diverse biological processes, including coagulation and immune regulation.^5^^,^^6^ Although initially associated with inflammatory and neoplastic diseases,^7^^,^^8^ emerging evidence suggests that FGL2 may also influence metabolic pathways. Its role in HSCR, particularly its effects on ENCCs, remains unexplored.

Previous studies have demonstrated FGL2’s pro-apoptotic effects in various pathological contexts. For example, in chronic viral infections and cancers, FGL2 induces apoptosis through paracrine mechanisms, contributing to CD8^+^ T cell exhaustion.^9^ In coronary artery disease, FGL2 promotes endothelial cell apoptosis, exacerbating vascular dysfunction.^10^ These findings highlight the diverse mechanisms by which *FGL2* regulates apoptosis and suggest its potential involvement in other diseases, including HSCR. Furthermore, recent evidence indicates that *FGL2* modulates metabolic processes, such as bile acid homeostasis, lipid metabolism, and cholesterol regulation, further linking it to metabolic disorders.^7^^,^^11^^,^^12^

Given the critical role of metabolism, particularly OXPHOS, in ENCC survival and differentiation, we hypothesize that FGL2 involved in HSCR pathogenesis by dysregulating ENCC metabolism. Specifically, we propose that FGL2 enhances OXPHOS mediated by JAK2-STAT3 pathway, leading to excessive reactive oxygen species (ROS) production, apoptosis, and impaired ENCC survival. To test this hypothesis, we conducted a series of *in vitro* experiments using ENCCs isolated from the intestines of embryonic mice obtained from pregnant mice, integrating transcriptomic profiling, biochemical assays, and functional analyses. In this study, we investigate the role of FGL2 as a metabolic regulator and its impact on ENCC function, providing insights into the metabolic basis of HSCR, and exploring the potential as a diagnostic marker was explored.

## Results

### Fibrinogen-like protein 2 is upregulated in aganglionic segment tissues

**Figure 1:**
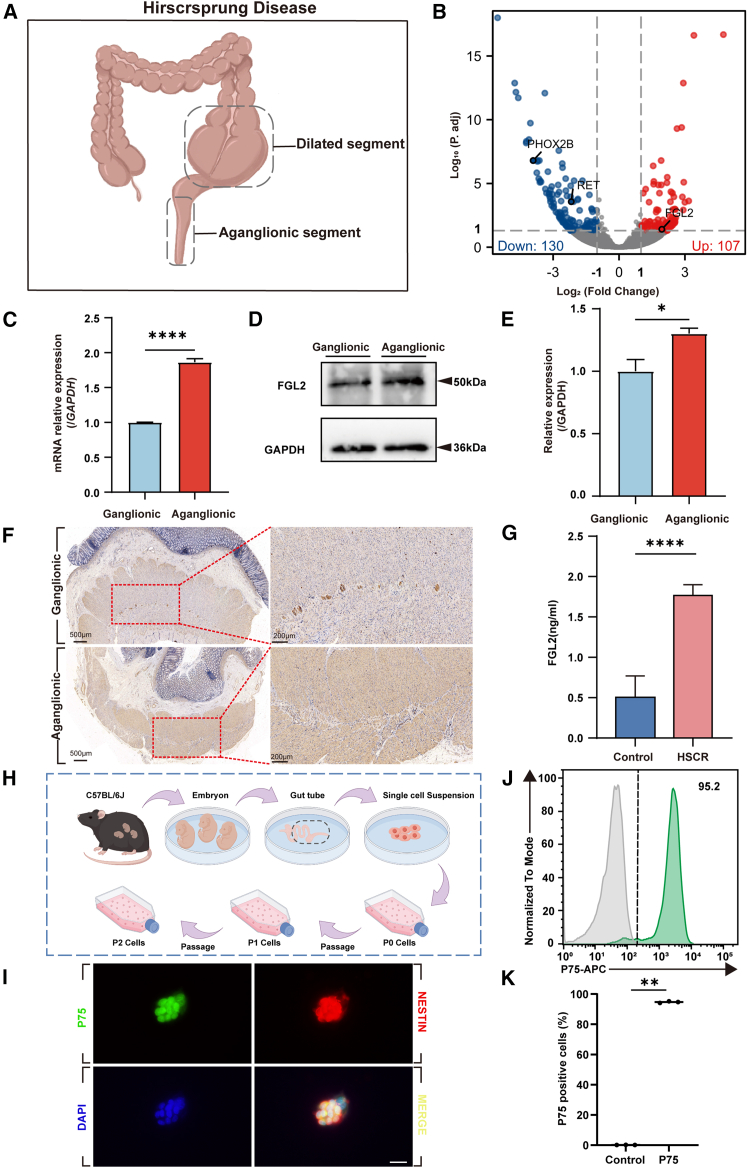
FGL2 is significantly upregulated in the aganglionic segment of HSCR (A) Schematic model of Hirschsprung disease tissue sample acquisition. (B) Volcano plot of DEGs from RNA-seq analysis of aganglionic and ganglionic segments of patients with HSCR. Red dots indicate significantly upregulated genes, and blue dots represent significantly downregulated genes in the aganglionic segment. (C) The RNA levels of *FGL2* were detected from aganglionic and ganglionic segments of patients with HSCR using qPCR. *GAPDH* was used as the control. *n* = 3 segments per group. Data are presented as mean ± SEM. Statistical analysis was performed using an unpaired two-tailed t-test. Significance reported as ∗∗∗∗*p* < 0.0001. (D) Western blot analysis of FGL2 protein in aganglionic and ganglionic segments of patients with HSCR, GAPDH was used as the loading control. *n* 3 segments per group. (E) The relative gray value was calculated by *ImageJ* software. Data are presented as mean ± SEM. Statistical analysis was performed using an unpaired two-tailed t-test. Significance reported as ∗*p* < 0.05. (F) IHC was conducted to assess the expression levels of FGL2 in aganglionic segment and ganglionic segment tissue of human tissue. Scale bar = 500 μm in left figure and Scale bar = 200 μm in right figure. *n* = 3 segments per group. (G) The serum FGL2 expression levels in experimental and control children were detected by ELISA. *n* = 6 patients per group. Data are represented as mean ± SEM. Statistical analysis was performed using unpaired two-tailed t-test. Significance reported as ∗∗∗∗*p* < 0.0001. (H) Schematic illustration of the procedure used to isolate enteric neural crest cells (ENCCs) from the gut of E14–15 C57BL/6J mouse embryos. (I) Immunofluorescence staining showing that the majority of isolated ENCCs were double-positive for P75 (green) and NESTIN (red), with nuclei counterstained using DAPI (blue). Scale bar = 20 μm. (J) Flow cytometry analysis of isolated ENCCs based on P75 surface marker expression. (K) Quantification of flow cytometry results confirming high purity of ENCC cultures. *n* = 3 segments per group. Data are presented as mean ± SEM. Statistical analysis was performed using an unpaired two-tailed t-test. Significance reported as ∗∗*p* < 0.01.

To identify key genes involved in the pathogenesis of HSCR, we performed RNA sequencing (RNA-seq) on six tissue samples from the aganglionic and ganglionic segments of three patients with HSCR (Figure 1A). Differential expression analysis revealed 237 differentially expressed genes (DEGs), with *FGL2* significantly upregulated in the aganglionic segment compared to the ganglionic segment (Figure 1B).

To validate these findings, we assessed *FGL2* expression using qRT-PCR and Western blot. Consistent with the RNA-seq results, qRT-PCR demonstrated a significant increase in *FGL2* mRNA levels in aganglionic tissues (Figure 1C), and Western blot confirmed the elevated protein expression of FGL2 in these samples (Figures 1D and 1E). Notably, in the ganglionic segment, FGL2 immunoreactivity appeared to be more concentrated in the myenteric plexus than in the surrounding muscular layer, suggesting that this spatial distribution pattern of FGL2 may reflect region-specific functional roles in ENS homeostasis (Figure 1F). ELISA assay revealed that the FGL2 level of HSCR was significantly elevated (Figure 1G).

These findings indicate that *FGL2* is upregulated in aganglionic tissues of HSCR, and the region-specific implicating it as a potential contributor to ENS.

### Fibrinogen-like protein 2 promotes apoptosis and suppresses the proliferation and migration of enteric neural crest cells

To investigate the role of FGL2 on ENCCs, we isolated primary ENCCs from the gut of E14-15 C57BL/6J mouse embryos (Figure 1H). Immunofluorescence staining confirmed that >90% of the cells were positive for both P75 and Nestin (Figure 1I), and flow cytometry further validated ENCC purity at 95.2% based on P75 expression (Figures 1J and 1K). Only cell batches with >95% immunofluorescence-confirmed P75 and Nestin expression were used for subsequent experiments.

**Figure 2:**
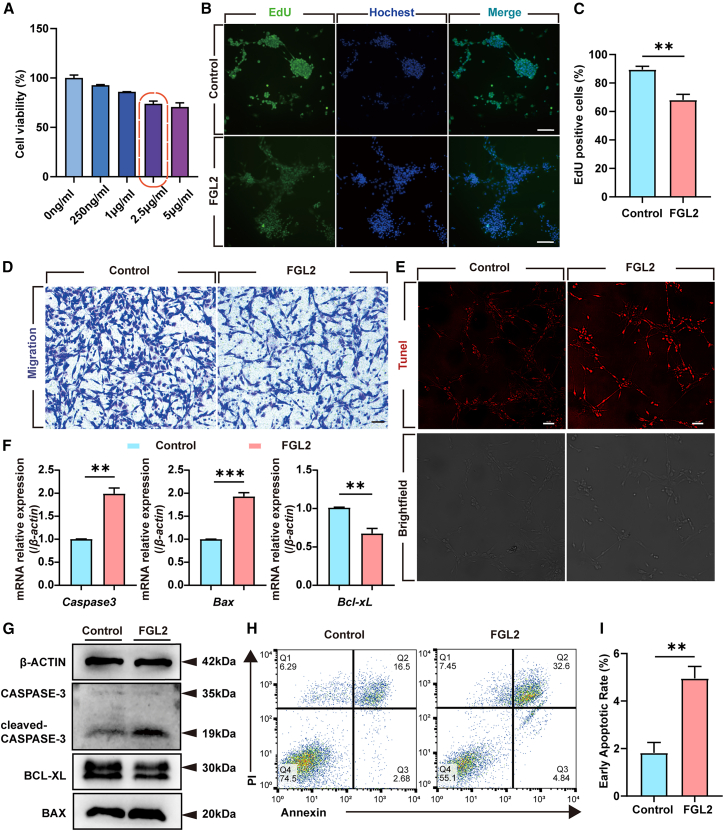
FGL2 induces apoptosis of ENCCs *in vitro* (A) Cell viability assessment of ENCCs treated with rFGL2 at different concentrations using CCK-8 assay. Results show a dose-dependent decrease in ENCC viability with rFGL2 treatment. *n* = 3 wells per group. (B) Proliferation analysis of ENCCs treated with rFGL2 using EdU assay. The percentage of EdU-positive cells (green) was significantly reduced in the FGL2-treated group compared to the control group. DAPI (blue) was used to stain nuclei. Scale bar = 100 μm. *n* = 3 wells per group. (C) Quantification of the percentage of EdU-positive cells was significantly reduced. Data are presented as mean ± SEM. Statistical analysis was performed using an unpaired two-tailed t-test. Significance reported as ∗∗*p* < 0.01. (D) Migration analysis of ENCCs treated with rFGL2 using Transwell assay. Scale bar = 50 μm. *n* = 3 wells per group. (E) Apoptosis detection in ENCCs treated with rFGL2 using TUNEL staining. The percentage of TUNEL-positive cells (red) was significantly increased in the FGL2-treated group. We also present bright-field images. Scale bar = 50 μm. *n* = 3 wells per group. (F) The RNA levels of apoptosis-related genes were detected using qPCR. The level of *Caspase-3* and *Bax* was significantly upregulated, and *Bcl-xL* was significantly reduced. *β-actin* was used as the control. *n* = 3 wells per group. Data are presented as mean ± SEM. Statistical analysis was performed using an unpaired two-tailed t-test. Significance reported as ∗∗*p* < 0.01, ∗∗∗*p* < 0.001. (G) Western blot analysis of apoptosis-related proteins in ENCCs treated with rFGL2. Cleaved-CASPASE3 and BAX expression levels were significantly upregulated, and BCL-XL expression level was significantly reduced in the FGL2-treated group compared to the control group. β-ACTIN was used as a loading control. *n* = 3 wells per group. (H) Flow cytometry analysis of apoptosis in ENCCs treated with rFGL2 using FITC/PI staining. The percentage of early and late apoptotic cells was significantly increased in the FGL2-treated group compared to the control group. *n* = 3 wells per group. (I) Quantification of early apoptotic ENCCs shows significant increase in the FGL2-treated group compared to the control. Data are represented as mean ± SEM. Statistical analysis was performed using unpaired two-tailed t-test. Significance reported as ∗∗*p* < 0.01.

To investigate the effects of FGL2 on ENCC viability, cells were stimulated with varying concentrations of recombinant FGL2 (rFGL2) and analyzed using the CCK-8 assay. rFGL2 treatment decreased ENCC viability in a dose-dependent manner (Figure 2A), with 2.5 μg/mL selected as the optimal concentration for subsequent experiments. EdU assays revealed reduced ENCC proliferation in the FGL2-treated group (Figures 2B and 2C), while Transwell assays showed significant inhibition of ENCC migration (Figure 2D).

To further explore the pro-apoptotic effects of FGL2, TUNEL staining revealed a marked increase in ENCC apoptosis after rFGL2 treatment (Figure 2E). Western blot and qRT-PCR analyses demonstrated the upregulation of *cleaved-Caspase-3* and *Bax*, and a decrease of *Bcl-xL* further supporting the induction of apoptosis (Figures 2F and 2G). Flow cytometry confirmed an increased percentage of early apoptotic cells in rFGL2-treated ENCCs (Figures 2H and 2I). Together, these findings indicate that FGL2 promotes apoptosis by inducing intrinsic apoptotic pathways in ENCCs.

### Fibrinogen-like protein 2 upregulates the oxidative phosphorylation pathway

**Figure 3:**
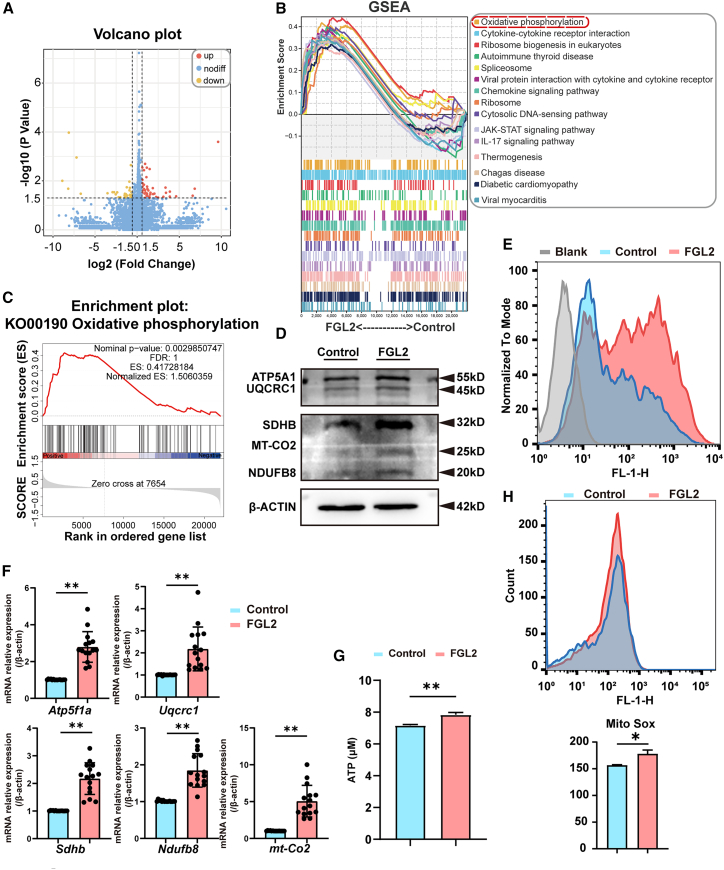
FGL2 treatment leads to mitochondrial dysfunction and increased ROS accumulation in ENCCs (A) Volcano plot of differentially expressed genes (DEGs) from RNA-seq analysis of FGL2-treated group compared to the control group. Red dots indicate significantly upregulated genes, and yellow dots represent significantly downregulated genes in FGL2 group. *n* = 5 wells per group. (B) GSEA of KEGG pathways analysis based on RNA-seq data. (C) Among the GSEA of KEGG pathways analysis, OXPHOS was the most enriched pathway in the FGL2 group. (D) Western blot analysis of OXPHOS-related proteins in ENCCs treated with rFGL2. ATP5A1, UQCRC1, SDHB, MT-CO2, and NDUFB8 expression levels were significantly upregulated in the FGL2-treated group compared to the control group. β-ACTIN was used as a loading control. *n* = 3 wells per group. (E) Flow cytometry analysis of intracellular ROS levels in ENCCs treated with rFGL2. ROS levels were significantly elevated in the FGL2-treated group compared to the control group. *n* = 3 wells per group. (F) The RNA levels of OXPHOS-related genes were detected using qPCR. The level of *Atp5f1a, Uqcrc1, Sdhb, mt-Co2*, and *Ndufb8* was significantly upregulated. *β-actin* was used as the control. *n* = 15 wells per group. Data are presented as mean ± SEM. Statistical analysis was performed using an unpaired two-tailed t-test. Significance reported as ∗∗*p* < 0.01. (G) Quantification of ATP levels in ENCCs. ATP production was significantly increased in the FGL2-treated group compared to the control group, indicating enhanced mitochondrial activity. Data are presented as mean ± SEM. Statistical analysis was performed using an unpaired two-tailed t-test. Significance reported as ∗∗*p* < 0.01. *n* = 3 wells per group. (H) mtROS levels in ENCCs treated with rFGL2 were quantified using MitoSOX staining and flow cytometry. The FGL2-treated group exhibited a significant increase in mtROS levels compared to the control group. *n* = 3 wells per group. Data are represented as mean ± SEM. Statistical analysis was performed using unpaired two-tailed t-test. Significance reported as ∗*p* < 0.05.

To elucidate the mechanism underlying FGL2-mediated ENCC apoptosis, RNA-seq was performed on ENCCs stimulated with or without rFGL2 (2.5 μg/ml). The analysis identified 76 DEGs (43 upregulated, 33 downregulated) in the FGL2-treated group compared to controls (Figure 3A). Gene set enrichment analysis (GSEA) of the RNA-seq data revealed significant upregulation of the OXPHOS pathway in rFGL2-stimulated ENCCs (Figures 3B and 3C).

qRT-PCR and Western blot further confirmed the upregulation of OXPHOS-related genes, including *Atp5a1, Uqcrc1, Sdhb, mt-Co2, and Ndufb8* (Figures 3D and 3F). And the normalized count values of RNA-seq for these five genes still showed an upward trend in the RNA-seq dataset (Figure S1).

Given OXPHOS is the process whereby cells use carbon fuels and oxygen to generate ATP, we further assessed the ATP levels to assess the functional impact of OXPHOS upregulation.^13^ The functional assessment of ATP levels revealed increased ATP production in rFGL2-treated ENCCs, indicating enhanced mitochondrial activity^14^ (Figure 3G).

Furthermore, as OXPHOS generates reactive oxygen species (ROS) as by-products.^14^ ROS levels were quantified by flow cytometry, which confirmed the elevated level of ROS production (Figure 3E). Given that mitochondria are a major source of ROS,^15^^,^^16^ and the mitochondrial respiratory chain, we further focused on measuring mitochondrial function in FGL2-treated ENCCs. Herein, the overproduction of mitochondrial ROS (mtROS) highlights the role of mtROS as a driving factor in the overall increase of ROS in the cell (Figure 3H). Together, these findings suggest that FGL2-induced OXPHOS upregulation promotes ROS accumulation, contributing to altered cellular metabolism and redox homeostasis in ENCCs.

### Mitochondrial dysfunction in fibrinogen-like protein 2-treated enteric neural crest cells is characterized by increased reactive oxygen species and altered membrane potential

**Figure 4:**
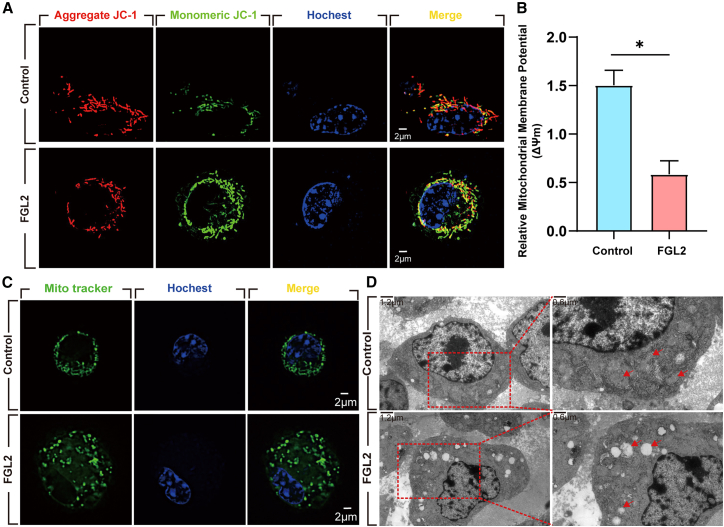
FGL2 enhances OXPHOS levels in ENCCs and increases mitochondrial activity (A) Mitochondrial membrane potential in ENCCs treated with rFGL2 was assessed using JC-1 staining. Scale bar = 2 μm. *n* = 3 wells per group. (B) Analysis of aggregate-to-monomer ratio in JC-1 assay. Data are presented as mean ± SEM. Statistical analysis was performed using an unpaired two-tailed t-test. Significance reported as ∗*p* < 0.05. (C) Mitochondrial morphology in ENCCs treated with rFGL2 visualized using MitoTracker staining. FGL2 treatment induced mitochondrial fission, indicating mitochondrial stress and damage. Scale bar = 2 μm *n* = 3 wells per group. (D) Ultrastructural analysis of ENCCs treated with rFGL2 using TEM. Mitochondrial swelling, cristae disruption, and vacuolation were observed in the FGL2-treated group, along with nuclear chromatin condensation, indicative of apoptosis. Scale bar = 1.2 μm in left figure and Scale bar = 0.6 μm in right figure. *n* = 3 wells per group.

Given the upregulation of OXPHOS, we further assessed mitochondrial function in rFGL2-treated ENCCs. JC-1 staining showed a reduced aggregate-to-monomer ratio, indicating a loss of mitochondrial membrane potential (MMP) and early mitochondrial dysfunction (Figures 4A and 4B).

MitoTracker assays revealed significant mitochondrial fission in rFGL2-treated ENCCs, consistent with mitochondrial damage (Figure 4C). Transmission electron microscopy (TEM) analysis further demonstrated mitochondrial swelling, fragmentation, and vacuolation, alongside nuclear chromatin condensation and margination—hallmarks of cellular stress and apoptosis (Figure 4D).

These results suggest that FGL2 induces mitochondrial dysfunction, contributing to ENCC apoptosis.

### Fibrinogen-like protein 2-induced oxidative phosphorylation elevation promotes enteric neural crest cell apoptosis via reactive oxygen species

**Figure 5:**
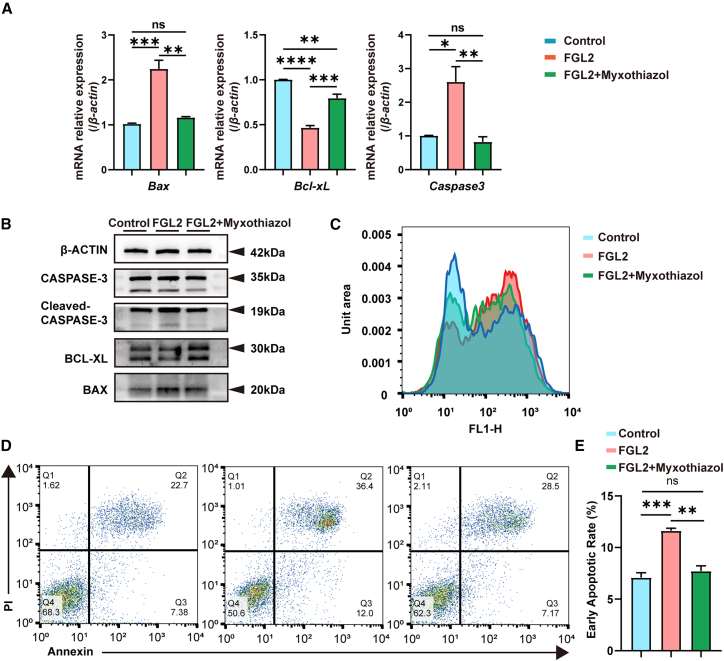
Pharmacological inhibition of OXPHOS attenuates FGL2-induced apoptosis in ENCCs (A) RNA levels of apoptosis-related genes were detected using qPCR. The RNA levels of *Cleaved-Caspase-3* and *Bax* were significantly upregulated and *Bcl-xL* expression level was significantly reduced in the FGL2-treated group compared to the control group, and were rescued by myxothiazol. *β-actin* was used as control. *n* = 3 wells per group. Data are presented as mean ± SEM. Statistical analysis was performed using an unpaired two-tailed t-test. Significance reported as ∗*p* < 0.05, ∗∗*p* < 0.01, ∗∗∗*p* < 0.001, ∗∗∗∗*p* < 0.0001, ns: no significance. (B) Western blot analysis of apoptosis-related proteins in ENCCs treated with rFGL2. Cleaved-CASPAS-3 and BAX expression levels were significantly upregulated and BCL-XL expression level was significantly reduced in the FGL2-treated group compared to the control group, and were rescued by myxothiazol. β-ACTIN was used as a loading control. *n* = 3 wells per group. (C) Flow cytometry analysis of intracellular ROS levels in ENCCs treated with rFGL2. ROS levels were significantly elevated in the FGL2-treated group compared to the control group, and were reduced by myxothiazol in FGL2-treated group. *n* = 3 wells per group. (D) Flow cytometry analysis of apoptosis in ENCCs treated with rFGL2 using FITC/PI staining. The percentage of early and late apoptotic cells was significantly increased in the FGL2-treated group compared to the control group, and was rescued by myxothiazol. *n* = 3 wells per group. (E) Quantification of early apoptotic ENCCs show significant increase in the FGL2-treated group compared to the control, and were rescued by myxothiazol. Data are represented as mean ± SEM. Statistical analysis was performed using unpaired two-tailed t-test. Significance reported as ∗∗*p* < 0.01, ∗∗∗*p* < 0.001, ns: no significance. *n* = 3 wells per group.

To explore the functional role of OXPHOS in FGL2-induced apoptosis, we treated rFGL2-stimulated ENCCs with Myxothiazol, a mitochondrial electron transport chain (ETC) complex III inhibitor. Myxothiazol, a mitochondrial electron transport chain (ETC) complex III inhibitor, is widely used in studies on OXPHOS regulation.^17^ Results showed that after adding Myxothiazol, the expression of apoptosis-related genes detected by qRT-PCR was significantly decreased, and Western blot analysis confirmed a reduction in the expression of apoptosis-related proteins (Figures 5A and 5B). Furthermore, flow cytometry analysis using an apoptosis detection kit demonstrated a notable decrease in the apoptosis ratio of ENCCs (Figures 5D and 5E). Furthermore, the ROS level was rescued by Myxothiazol (Figure 5C). These findings suggest that FGL2 promotes ENCC apoptosis by upregulating OXPHOS activity.

**Figure 6:**
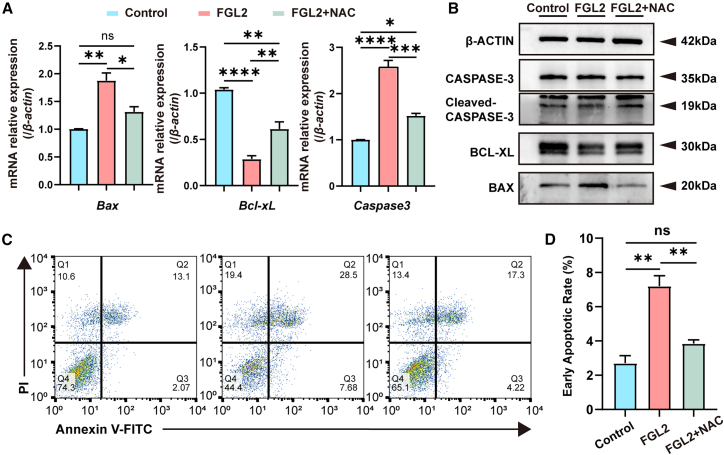
Pharmacological inhibition of ROS attenuates FGL2-induced apoptosis in ENCCs (A) RNA levels of apoptosis related genes were detected using qPCR. The RNA levels of *Cleaved-Caspase-3* and *Bax* were significantly upregulated and *Bcl-xL* expression level was significantly reduced in the FGL2-treated group compared to the control group, and were rescued by NAC in FGL2-treated group. *β-actin* was used as control. Data are presented as mean ± SEM. Statistical analysis was performed using an unpaired two-tailed t-test. Significance reported as ∗*p* < 0.05, ∗∗*p* < 0.01, ∗∗∗*p* < 0.001, ∗∗∗∗*p* < 0.0001, ns: no significance. *n* = 3 wells per group. (B) Western blot analysis of apoptosis-related proteins in ENCCs treated with rFGL2. Cleaved-CASPASE-3 and BAX expression levels were significantly upregulated and BCL-XL expression level was significantly reduced in the FGL2-treated group compared to the control group, and were rescued by NAC in FGL2-treated group. β-ACTIN was used as a loading control. *n* = 3 wells per group. (C) Flow cytometry analysis of apoptosis in ENCCs treated with rFGL2 using FITC/PI staining. The percentage of early and late apoptotic cells was significantly increased in the FGL2-treated group compared to the control group, and was rescued by NAC in FGL2-treated group. *n* = 3 wells per group. (D) Quantification of early apoptotic ENCCs shows significant increase in the FGL2-treated group compared to the control, and were rescued by NAC in FGL2-treated group. *n* = 3 wells per group. Data are represented as mean ± SEM. Statistical analysis was performed using unpaired two-tailed t-test. Significance reported as ∗∗*p* < 0.01, ns: no significance.

To determine whether ROS plays a central role, we treated rFGL2-stimulated ENCCs with N-acetylcysteine (NAC), a ROS inhibitor. NAC (2 mM)^18^ treatment significantly suppressed the expression of apoptosis-related genes and proteins, as well as the apoptosis ratio (Figures 6A–6D).

### Fibrinogen-like protein 2 activates the JAK2–STAT3 signaling pathway to promote oxidative phosphorylation in enteric neural crest cell

**Figure 7:**
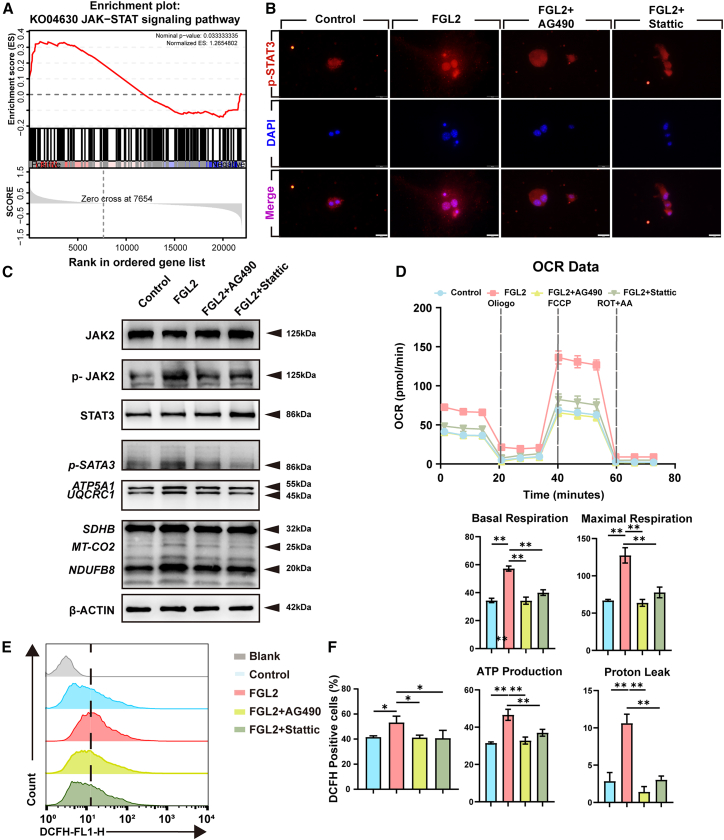
FGL2 enhances OXPHOS via the JAK2–STAT3 signaling pathway in ENCCs (A) Gene Set Enrichment Analysis (GSEA) of RNA-seq data from ENCCs treated with rFGL2 (2.5 μg/mL, 6 h) revealed the upregulation of the JAK-STAT signaling pathway. (B) Immunofluorescence staining showed the nuclear accumulation of phosphorylated STAT3 (p-STAT3) upon FGL2 stimulation, which was reduced by pretreatment with JAK2 inhibitor AG490 (50 μM) or STAT3 inhibitor Stattic (5 μM). Scale bar = 20 μm. *n* = 3 wells per group. (C) Western blot analysis of phosphorylated and total JAK2 and STAT3 in ENCCs treated with FGL2, AG490, or Stattic. Quantification confirmed the activation of JAK2–STAT3 by FGL2 and suppression by respective inhibitors. Data are presented as mean ± SEM, *n* = 3 wells per group. Western blot analysis of representative OXPHOS complex proteins (ATP5A1, UQCRC1, SDHB, MTCO2, NDUFB8) revealed increased expression after FGL2 treatment, attenuated by JAK2 or STAT3 inhibition. β-ACTIN was used as a loading control. *n* = 3 wells per group. (D) Seahorse XF Cell Mito Stress Test showed increased oxygen consumption rate (OCR) in ENCCs following FGL2 treatment, which was significantly reversed by the inhibition of JAK2 or STAT3. Basal respiration, maximal respiration, spare respiratory capacity, and proton leak were quantified, *n* = 3 wells per group. (E and F) Flow cytometry detection of intracellular ROS levels (DCFH-DA staining) indicated that FGL2-induced ROS accumulation was reduced upon treatment with AG490 or Stattic. Data are presented as mean ± SEM. Statistical analysis was performed using an unpaired two-tailed t-test. Significance reported as ∗*p* < 0.05, ∗∗*p* < 0.01. *n* = 3 wells per group.

To investigate the molecular mechanism by which FGL2 modulates OXPHOS in ENCCs, we further performed the analysis of RNA-seq in ENCCs treated with FGL2, and the GSEA result revealed the upregulation of the JAK-STAT signaling pathway (Figure 7A). Among all STAT isoforms, STAT3 was uniquely reported to localize to mitochondria and regulate mitochondrial respiration.^19^ Since JAK2 is a canonical upstream kinase of STAT3, we further tested the activation of the JAK2-STAT3 pathway upon FGL2 stimulation.^20^

Western blot analysis demonstrated that FGL2 significantly increased phosphorylation levels of JAK2 and STAT3 in ENCCs, providing direct evidence for the activation of the JAK2-STAT3 pathway. The application of a JAK2-specific inhibitor reduced STAT3 phosphorylation, whereas STAT3 inhibition did not affect JAK2 phosphorylation, confirming the upstream–downstream relationship (Figure 7C). Furthermore, FGL2 significantly increased phosphorylated STAT3 (pSTAT3) in the nucleus as evidenced by immunofluorescent analyses. And pharmacological blockade of JAK2 (50 μM AG490) or STAT3 (5 μM Stattic)^21^^,^^22^ dramatically attenuated FGL2-induced nuclear accumulation (Figure 7B).

We next examined the effect of JAK2-STAT3 pathway on mitochondrial function. Treatment with either JAK2 or STAT3 inhibitors significantly decreased OXPHOS complex protein levels and intracellular ROS accumulation. Seahorse assay further demonstrated that FGL2 enhanced the oxygen consumption rate (OCR), which was significantly attenuated by JAK2 or STAT3 inhibition (Figure 7D). The basal respiration capacity was elevated prominently by FGL2 and weakened by the stimulation of AG490 or Stattic. The maximal respiration capacity and spare respiratory capacity were also elevated in the FGL2-treated group, suggesting that FGL2 promotes a more active mitochondrial respiratory state and is inhibited by JAK2-STAT3 axis. Additionally, an increase in proton leak suggests a potential loss of mitochondrial efficiency or an increase in mitochondrial stress, and the status could be reversed by a JAK2-STAT3 inhibitor. In addition, ROS production could be decreased by the JAK2-STAT3 inhibitor (Figures 7E and 7F). These results suggest that FGL2 enhances OXPHOS in ENCCs by activating the JAK2-STAT3 signaling axis.

Together, these findings demonstrate that FGL2 promotes ENCC apoptosis by modulating OXPHOS-induced ROS overproduction through JAK2-STAT3 signaling axis. This study provides mechanistic evidence supporting the role of OXPHOS in the pathogenesis of HSCR mediated by FGL2.

## Discussion

HSCR remains a significant clinical challenge due to its complex pathogenesis and potential for recurrence. Characterized by the absence of ENCCs in the distal gut, HSCR arises from defects in ENCC growth, migration, and differentiation, leading to intestinal obstruction.^2^ While prior research has identified several pathogenic genes, including *RET* and *EDNRB*,^1^ the role of *FGL2* in HSCR pathogenesis has not been well understood. This study provides evidence implicating FGL2 as a critical factor in HSCR, acting through its effects on mitochondrial function and metabolism to drive ENCC apoptosis. While our findings demonstrate that FGL2 promotes ENCC apoptosis via mitochondrial OXPHOS and ROS generation *in vitro*, we acknowledge that this does not fully establish causality in the *in vivo* setting. Our experimental analysis of FGL2’s functional interactions within ENCCs revealed compelling evidence suggesting that FGL2 upregulation may function as a pathogenic driver in HSCR.

RNA sequencing identified *FGL2* as significantly upregulated in the aganglionic segments of HSCR, consistent with previous studies highlighting regional heterogeneity in gene expression.^23^^,^^24^ Interestingly, we observed that in the ganglionic segment, FGL2 was more prominently expressed in the myenteric plexus, rather than diffusely distributed throughout the muscular layer. This localized expression pattern may reflect FGL2’s role in enteric neuronal maintenance or communication, and warrants further investigation into its spatial dynamics within the enteric nervous system.

Functional assays revealed that FGL2 impairs ENCC proliferation and migration while promoting apoptosis. Interestingly, while *FGL2* has been primarily studied in cancer and inflammatory diseases,^6^^,^^7^ its pro-apoptotic role in ENCCs contrasts with its tumor-promoting effects in cancers such as hepatomas and brain tumors.^25^^,^^26^ This underscores the multifaceted nature of *FGL2*, whose functions appear to be highly context-dependent.

The pro-apoptotic effects of FGL2 in ENCCs are reminiscent of its homolog *FGL1*, which regulates immune escape via *LAG-3* receptor activation.^27^ In hepatocellular carcinoma, *FGL1* demonstrates dual roles, suppressing tumor cell proliferation while promoting immune evasion.^6^ Similarly, our findings highlight a dual role for FGL2 in HSCR, with its pro-apoptotic effects contributing to ENCC loss and impaired gut innervation. Future research should explore potential interactions between FGL2 and other signaling pathways to further delineate its role in ENCC biology.

Our study reveals a metabolic mechanism by which FGL2 influences HSCR pathogenesis. FGL2 upregulates OXPHOS in ENCCs, resulting in excessive ROS production and subsequent apoptosis. This finding aligns with prior research linking OXPHOS dysregulation to impaired ENCC survival and differentiation in HSCR.^2^^,^^4^ Importantly, we demonstrate that OXPHOS inhibition significantly rescues ENCC apoptosis, confirming its central role in mediating FGL2-induced cellular dysfunction.

ROS, as natural byproducts of OXPHOS, play a dual role in cellular signaling and stress responses.^28^ While physiological levels of ROS are critical for normal cellular processes, excessive ROS can lead to oxidative damage, mitochondrial dysfunction, and programmed cell death.^14^^,^^29^ In our study, mitochondrial ROS (mtROS) emerged as a key driver of ENCC apoptosis following FGL2 stimulation, highlighting the importance of mitochondrial integrity in ENCC survival. Consistent with previous reports, we observed mitochondrial swelling, cristae loss, and membrane potential disruption in ENCCs, further linking mitochondrial dysfunction to HSCR pathogenesis.^30^

The interplay between metabolic regulation and ENCC apoptosis sheds light on the mechanisms underlying HSCR. Alterations in OXPHOS activity, whether excessive or insufficient, appear to compromise ENCC survival and differentiation. These findings emphasize the need for finely tuned OXPHOS activity to maintain cellular homeostasis. By identifying FGL2 as a modulator of OXPHOS and ROS production, this study opens avenues for therapeutic exploration, such as targeting FGL2 or OXPHOS pathways to mitigate ENCC loss in HSCR.

Mechanically, this finding demonstrated that FGL2 modulates mitochondrial metabolism in ENCCs through the JAK2-STAT3 signaling pathway, leading to OXPHOS enhancement and oxidative stress-induced apoptosis. This signaling axis may represent a mechanistic link between extracellular FGL2 and intracellular metabolic remodeling, providing insights into the regulation of ENCC survival in HSCR.

Emerging evidence indicates that macrophage-derived FGL2 secretory protein plays significant roles in multiple pathological contexts, including alcoholic liver injury^31^ and melanoma pathogenesis.^32^ Notably, studies in intestine, particularly in inflammatory bowel disease (IBD),^11^ have corroborated the macrophage origin of FGL2 during colonic inflammation. Building upon these observations, we hypothesize that in HSCR, the apoptosis-inducing FGL2 affecting ENCCs may originate from infiltrating macrophages within the aganglionic intestinal segments. To validate this mechanistic link and spatially resolve cellular interactions, subsequent studies need to utilize single-cell RNA sequencing.

In summary, this study identifies FGL2 as a regulator of ENCC metabolism and apoptosis in HSCR. By upregulating OXPHOS and ROS production, FGL2 disrupts ENCC survival, highlighting mitochondrial dysfunction as a central mechanism in HSCR pathogenesis. These findings contribute to a growing body of evidence linking metabolic dysregulation to developmental disorders and provide a foundation for future exploration into the broader roles of FGL2 in gut development and disease mechanisms.

### Limitations of the study

While our study provides compelling *in vitro* evidence that FGL2 promotes ENCC apoptosis via OXPHOS upregulation and ROS accumulation, we acknowledge that the current findings are limited to primary mouse-derived ENCCs. The lack of *in vivo* validation restricts the generalizability of the proposed mechanism. Future studies should employ conditional FGL2 knockout or overexpression mouse models, as well as human-derived intestinal organoids, to determine the upregulation of FGL2 was the cause or compensation and to more accurately assess the physiological relevance of the FGL2–OXPHOS–ROS axis in HSCR development. Besides, the relatively small sample size may limit the generalizability of the findings, underscoring the need for validation in larger cohorts of patients with HSCR.

## Resource availability

### Lead contact

Further information and requests for resources and reagents should be directed to and will be fulfilled by the Lead contact, Aiwu Li (liaiwu@qiluhospital.com).

### Materials availability

Reagents generated in this study will be made available by reasonable request to the lead contact.

### Data and code availability

• Bulk RNA-seq data generated in this study have been deposited in Gene Expression Omnibus (GEO) database, respectively, and are publicly available as of the date of publication. Accession numbers are listed in the key resources table.
• This study does not report original code.
• Any additional information required to reanalyze the data reported in this article is available from the lead contact upon request.

## Acknowledgments

We appreciate the technical support from the Basic Medical Research Center of Qilu Hospital, Shandong University. This research was funded by the 10.13039/501100001809National Natural Science Foundation of China (No. 82271743, 82071682), the Taishan Scholar Foundation of Shandong Province (award number tstp20221155), the Cheeloo Medical Development Fund of Shandong University (34641390220001), the 10.13039/501100007129Natural Science Foundation of Shandong Province (No. ZR2022MH276, ZR2021MH210, ZR2021MH334), and Natural Science Foundation Project of Hunan Province (2024JJ7555).

## Author contributions

Jichang Han, Xiaoyang Liu, and Yixuan Wang performed most of the experiments, analyzed data, and wrote the article draft. Qiongqian Xu, Dong Sun, Xintao Zhang, and Xixi He contributed to the data analysis and provided technical support. Chuncan Ma and Xue Ren assisted with methodology optimization and experimental validation. Jian Wang, Yaru Mou, and Qiangye Zhang participated in sample collection and clinical data interpretation. Dongming Wang, Weijing Mu, and Peimin Hou supervised specific experiments and contributed to result interpretation. Aiwu Li conceived and supervised the project, acquired funding, and revised the article. All authors read and approved the final article.

## Declaration of interests

The authors declare no competing interests.

## STAR★Methods

### Key resources table

**Table:** 

REAGENT or RESOURCE	SOURCE	IDENTIFIER
**Antibodies**		

Anti-mouse FGL2 monoclonal antibody (M01), clone 6D9	Abnova	Cat#H00010875-M01; RRID: AB_565732
Anti-Rabbit Anti-GAPDH Monoclonal Antibody, Unconjugated, Clone 14C10	Cell Signaling Technology	Cat#2118; RRID: AB_561053
Anti-rabbit Caspase-3 (D3R6Y) Rabbit mAb	Cell Signaling Technology	Cat#14220; RRID: AB_2798429
Anti-rabbit Bax Antibody	Cell Signaling Technology	Cat# 2772; RRID: AB_10695870
Anti-rabbit Bcl-xL (54H6) Rabbit mAb	Cell Signaling Technology	Cat# 2764; RRID: AB_2228008
Anti-rabbit Beta Actin antibody	Proteintech	Cat# 20536-1-AP; RRID: AB_10700003
Anti-rabbit ATP5A1 Polyclonal antibody	Proteintech	Cat#14676-1-AP; RRID AB_2061761
Anti-rabbit UQCRC1 Polyclonal antibody	Proteintech	Cat#21705-1-AP; RRID AB_10734437
Anti-rabbit SDHB Polyclonal antibody	Proteintech	Cat#10620-1-AP; RRID AB_2285522
Anti-rabbit MTCO2 Polyclonal antibody	Proteintech	Cat#55070-1-AP; RRID AB_10859832
Anti-rabbit NDUFB8 Polyclonal antibody	Proteintech	Cat#14794-1-AP; RRID AB_2150970
Anti-rabbit Jak2 (D2E12) XP® Rabbit mAb	Cell Signaling Technology	Cat# 3230; RRID: AB_2128522
Anti-rabbit Phospho-Jak2 (Tyr1007/1008) Antibody	Cell Signaling Technology	Cat# 3771; RRID: AB_330403
Anti-rabbit Stat3 (D3Z2G) Rabbit mAb	Cell Signaling Technology	Cat# 12640; RRID: AB_2629499
Anti-rabbit Phospho-Stat3 (Tyr705) (D3A7) XP Rabbit mAb	Cell Signaling Technology	Cat# 9145; RRID: AB_2491009
Anti-rabbit p75 NGF Receptor antibody [EP1039Y]	Abcam	Cat# ab52987; RRID: AB_881682
Anti-rat Anti-Nestin Monoclonal Antibody, Unconjugated, Clone 7A3	Abcam	Cat# ab81462; RRID: AB_1640724
CD271 (NGF Receptor) Monoclonal Antibody (ME20.4), APC, eBioscience	Thermo Fisher Scientific	Cat# 17-9400-42; RRID: AB_2784656

**Biological samples**		

Human: conlon tissue of patients with HSCR	Qilu hospital of Shandong University	See Table S1
Human: serum of patients with HSCR or other control patients	Qilu hospital of Shandong University	See Table S1

**Chemicals, peptides, and recombinant proteins**		

Hoechst Staining Kit	Thermo Fisher Scientific	Cat#62249
DAPI	Beyotime	Cat#C1002
Mitochondrial Membrane Potential Assay Kit (JC-1)	Beyotime	Cat#C2006
MitoTracker	Beyotime	Cat#C1029
Seahorse XF Cell Mito Stress Test Kit	Agilent	Cat#103015-100
BCA Protein Assay Kit	Solarbio	Cat#PC0020
SDS-PAGE Sample Loading Buffer	Beyotime	Cat#P0015L
Enhanced Chemiluminescence Detection Kit	Millipore	Cat#WBKLS0100
PVDF Membrane	Millipore	Cat#98311
Protein Marker	Thermo Fisher Scientific	Cat#26616
FITC Annexin-V Apoptosis Detection Kit	BD Biosciences	Cat#556547
Tween 20	Solarbio	Cat#T8220
ATP Colorimetric Assay Kit	Beyotime	Cat#S0026
ROS Assay Kit	Beyotime	Cat#0033S
MitoSOX Red Mitochondrial Superoxide Indicator	Thermo Fisher Scientific	Cat#M36007
Cell Counting Kit-8 (CCK-8)	Yeasen	Cat#40203ES60
EdU Imaging Kit	RiboBio	Cat#C10310-1
One-Step TUNEL Apoptosis Assay Kit	Beyotime	Cat#C1086
SYBR Green Reagent	Toyobo	Cat#QPK-201
Methanol	Sinopharm Chemical Reagent Co.	Cat#67-56-1
Absolute Ethanol	Sinopharm Chemical Reagent Co.	Cat#64-17-5
Glutaraldehyde Fixative Solution	Macklin	Cat#G916055-500 mL
Isopropanol	Sinopharm Chemical Reagent Co.	Cat#80109218
Skim Milk Powder	BioFroxx	Cat#1172
WB Primary Antibody Dilution Buffer	Beyotime	Cat#P0023A
RIPA Lysis Buffer (High Efficiency)	Solarbio	Cat#R0010
Rapid Total RNA Extraction Kit	FlyGene	Cat#220010
Reverse Transcription Kit	Vazyme	Cat#R323-01
Goat Anti-Rabbit IgG (Alexa Fluor 488)	Proteintech	Cat#SA00006-2
Goat Anti-Mouse IgG (Alexa Fluor 594)	Proteintech	Cat#SA00006-3
Penicillin-Streptomycin (10×)	Gibco	Cat#15070063
0.25% Trypsin-EDTA	Gibco	Cat#25200056
4% Paraformaldehyde Fixative Solution	Beyotime	Cat#P0099-100 mL
TRIzol Reagent	Invitrogen	Cat#15596-018
Sterile Centrifuge Tubes	Corning	N/A
0.22 μm Cell Filter	Millipore	Cat#SLVV033RS
ChamQ Universal SYBR qPCR Master Mix	Yeasen	Cat#Q711
Cell Culture Flasks	Corning	N/A
5× Loading Buffer	CWBIO	Cat#CW0027S
Ammonium Oxalate-Crystal Violet Staining Solution (0.1%)	Solarbio	Cat#G1064
20× TBST Buffer	Solarbio	Cat#T1082
35 mm Confocal-Suitable Culture Dish	NEST Biotechnology	Cat#801001
Complete Medium for Mouse ENCCs	Annoron	Cat#CP-M216
Triton X-100	Solarbio	Cat#T8200
Ag490	Beyotime	Cat#S1509
Stattic	Abcam	Cat#ab120952

**Critical commercial assays**		

FGL2 ELISA Kit	AssayGenie	Cat#HUEB0701

**Deposited data**		

RNA-seq	This paper	GEO: GSE305450
RNA-seq	This paper	GEO: GSE305114.

**Experimental models: Organisms/strains**		

C57BL/6J mouse	SPF (BEIJING)BIOTECHNOLOGY Co., Ltd.	B2044

**Oligonucleotides**		

See Table S2 for qRT-PCR	This paper	N/A

**Software and algorithms**		

FlowJo version 10.4.2	FlowJo	https://www.flowjo.com
Prism 8.0.1	GraphPad Software	https://www.graphpad.com/scientific-software/prism/
ImageJ 1.52v	NIH	https://fiji.sc/
Imaris Viewer 9.5.1	Oxford Instruments Group	https://imaris.oxinst.com/
OlympusOlyVIA3.2.1	OLYMPUS	https://www.olympus-lifescience.com/en/discovery/image-sharing-made-easy meet-olyvia/

### Experimental model and subject details

#### Patients

A total of 21 patients participated in this study (Table S3). Among the patients, 15 patients were HSCR, 2 patients were cryptorchidism, 3 patients were hernia and one patient was ureteral calculus. The HSCR patients were ethnic Chinese subjects collected from Qilu Hospital of Shandong University from 2023 to 2024. The study protocol was approved by Qilu Hospital of Shandong University Institutional Review Board (Approval No. KYLL-2022(ZM)-192). This study was conducted according to government policies and the Helsinki Declaration. Written informed consent was obtained from all participants or their guardians prior to inclusion in the study.

Sporadic HSCR patients were recruited, and their biopsy specimens and surgical materials were reviewed by at least two independent pathologists. The diagnosis of HSCR was confirmed through histological examination, characterized by the absence of ganglion cells in both the myenteric and submucosal plexuses. According to the Chinese guidelines, patients representing all forms of HSCR—short-segment, common-segment, long-segment, and total colonic aganglionosis (TCA)—were included. Participants with additional congenital anomalies were excluded from the study.

Surgical specimens were collected from the dilated segment (proximal segment with ganglion cells) and aganglionic segment (distal segment without ganglion cells) of each patient. Each tissue sample weighed no less than 500 mg. A 0.5 cm portion of each sample was fixed in 4% paraformaldehyde for subsequent histological analysis, and the remainder was stored at −80°C.

### Method details

#### Cell culture

C57BL/6J mice at gestational days 14–15 were euthanized by cervical dislocation following ethical guidelines approved by the Institutional Animal Care and Use Committee (IACUC). Immediately after euthanasia, the mice were placed in 75% ethanol for a few minutes to ensure surface sterilization, and then soaked in sterile PBS to remove alcohol. Embryos of pregnant mice were aseptically removed, washed with PBS containing 1% penicillin-streptomycin, and then the gut tubes were dissected. The gut tubes were digested with 0.25% trypsin-EDTA solution at 37°C for 5–7 min. The separated cells were filtered through a sterile cell strainer with a pore size of 70 μm, followed by centrifugation at 1000 rpm for 5 min. The cells were collected and placed in culture flasks (Koning, New York, USA) containing complete mouse enteric neural crest cell culture medium (PromoCell, CP-M216) and maintained at 37°C with 5% CO2. The medium was half-changed every 24 h. The cells were passaged when reached 80–90% confluence. After 2–3 passages, immunofluorescence identification of P75 and nestin expression was performed, and the expression reached over 90%. Each batch of cells had to exhibit P75 and nestin expression of over 95% for subsequent cell experiments.

#### RNA extraction and quantitative PCR

Total RNA was isolated from colonic tissue of HSCR or ENCC using RNAfast2000 (Fastagen, China) and reverse-transcribed using a HiScript III RT SuperMix for qPCR kit (TOYOBO, Cat FSQ-101) according to the manufacturer’s protocol. All qRT-PCR reactions were run on an Applied Biosystems StepOne Real-Time PCR System (Thermo). *Gapdh/β-actin* was used as housekeeping gene to normalize the gene expression data across different samples. For analysis, the 2-ΔΔCt was used to calculate the relative mRNA expression of target genes.

The primers used are shown in Table S2.

#### Western blot

Colonic tissues and cells were lysed in radioimmunoprecipitation assay (RIPA) buffer supplemented with protease inhibitors (Roche) and phosphatase inhibitors (Beyotime). The protein concentration was determined using a BCA protein assay kit (CWBIO, China). Equal amounts of protein were subjected to electrophoresis on 10% sodium dodecyl sulfate-polyacrylamide gels (SDS-PAGE) and then transferred to polyvinylidene difluoride membranes (PVDF, Merck Millipore). The membranes were blocked with 5% bovine serum albumin (BSA) and 0.1% Tween 20 in Tris-buffered saline 1 h, and then incubated overnight at 4 °C with primary antibody. After washing with TBST, the appropriate horseradish peroxidase (HRP)-coupled secondary antibody was then added at 37 °C for 1 h, and was detected with chemiluminescent substrate BrightTM enhanced chemiluminescent (ECL) (Beyotime). The protein bands on the PVDF membranes were observed using an enhanced chemiluminescence kit (Millipore, USA) and quantified using ImageJ. The antibody used in this study is listed in Table S1 in the Supporting Information.

#### Immunohistochemistry (IHC)

Human conlon samples were decalcified, dehydrated, cleared with dimethylbenzene after fixation in 4% paraformaldehyde, and specimens were embedded in paraffin. Each slice was cut into 5-μm thick sections, which were pretreated with antigen retrieval buffer (enzymatic digestion) (AR0022; Boster Biological Technology, Wuhan, China) for 30 min at 37°C. After blocking in goat serum for 30 min at room temperature, serial slices were incubated with primary antibodies at 4°C overnight, followed by incubation with a horseradish peroxidase-conjugated secondary antibody for 60 min at room temperature. Add DAB solution, counterstain with hematoxylin, and observe positive staining under microscope.

#### Mitochondrial membrane potential detection probe

Mitochondrial membrane potential detection with Enhanced Mitochondrial Membrane Potential Detection Kit (JC-1, Cat#C2003S, Beyotime, China). ENCCs were cultured in confocal dishes. Incubate the ENCCs at 37 °C for 20 min with JC-1 working solution. After incubation, wash the samples twice with JC-1 staining buffer. Apply Hoechst (Beyotime Biotechnology, Cat#C1029, Shanghai, China) for 10 min at 37 °C, and observe them under the laser confocal microscope.

#### Measurement of intracellular ROS

The level of ROS was measured with ROS Assay Kit (Beyotime Biotechnology, Cat#0033S, Shanghai, China) by flow cytometry. ENCCs were incubated with DCFH-DA 10 μM, for 20 min at 37 °C. The stained cells were then washed with PBS and analyzed in flow cytometer (BD FACS Calibur, USA).

#### Mitochondrial ROS measurement

The level of mitochondrial ROS was measured with MitoSOX (Thermo Fisher Scientific, Cat#M36007, Sunnyvale, CA, USA) by flow cytometry. Cells were incubated with MitoSOX 1 μM for 30 min at 37°C and 5% CO2, washed with PBS 3 times and analyzed in flow cytometer (BD FACS Calibur, USA).

#### ATP measurement

ATP was measured using a bioluminescence assay kit (Beyotime Biotechnology, Cat#S0026, Shanghai, China). Briefly, the ENCCs were lysed in the lysis buffer provided with the kit. The supernatant was collected by centrifugation at 12,000 rpm for 5 min at 4 °C. The concentration of ATP present in the samples was determined by mixing 20 μL of the supernatant with 100 μL of luciferase reagent; the luciferase present in the reagent catalyzes the production of luminescence from ATP and luciferin. The luminescence of each sample was measured on a Infinte E plex microplate luminometer (Tecan, Mannedorf, Switzerland). A standard curve of ATP was prepared using a series of standards of known concentrations; the measured ATP is presented as nmol/ml.

#### ELISA

The blood serum was maintained in a freezer at −20°C until measurements. HSCR patients were FGL2group, and hernia patients were considered as control group. The levels of FGL2 were assayed through ELISA kit (AssayGenie, Cat#HUEB0701) according to the manufacturer’s instructions.

#### Transmission electron microscopy (TEM)

The ENCCs were collected by trypsinization, transferred into 2 mL centrifuge tubes and fixed with fixation solution (Macklin, Cat#G916055) for 2 h at 4°C. The cells were post-fixed in 1% osmium tetroxide in 0.1 M phosphate buffer (pH 7.4) for 2 h at room temperature (20°C). Afterward, the ENCCs were dehydrated in a graded ethanol series (50%, 70%, 80%, 90%, 95%, 100%, 100%) for 15 min for each solution and infiltrated with propylene oxide to embedding medium overnight. Ultrathin sections (50 nm) were obtained using an EM UC7 ultramicrotome (Leica), post-stained with uranyl acetate and lead citrate, and visualized using a transmission electron microscope (JEM-1200EX, JEOL, Tokyo, Japan).

#### Cell viability assays

Cell viability was determined using the CCK-8 assay with Cell Count Kit-8 (Yesen, Cat#40203ES60) following manufacturer’s instructions. Briefly, cells were seeded onto 96-well plates at a density of 6 × 103 cells/well. Cells were treated with rFGL2 at indicated concentrations for 6 h and subsequently incubated with CCK-8 reagent at 37°C for 2 h. The absorbance at 450 nm was measured on a Infinte E plex microplate luminometer (Tecan, Mannedorf, Switzerland).

#### Cell proliferation assay

ENCC proliferation was measured using Cell Proliferation EdU Image Kit (Abbkine, Wuhan, China) following manufacturer’s instructions. ENCCs were seeded in a 96-well plate at a density of 8000 cells per well. Cells were cultured overnight under standard culture conditions (37°C, 5% CO_2_) to allow adherence. Add EdU solution to the culture medium at a final concentration of 20 μM to ensure a final EdU concentration of 10 μM in the 96-well plate. Incubate the cells with EdU for 2 h in a humidified incubator at 37°C, 5% CO_2_. Wash the cells twice with PBS buffer to remove the medium. Fix the cells with 4% paraformaldehyde (0.1 mL per well) for 15 min at room temperature. Rinse the cells with 0.1 mL of BSA Wash Solution (1×) for 5 min, repeating this step three times to remove the fixative. Permeabilize the cells by adding 0.1 mL of PBS buffer containing 0.5% Triton X-100 for 15 min at room temperature. Wash the cells again with 0.1 mL of BSA Wash Solution (1×) for 5 min, repeating this step twice. Add 100 μL of the Click-iT reaction mixture to each well, containing an azide-labeled fluorescent dye (e.g., Alexa Fluor 488). Incubate the cells for 30 min at room temperature in the dark to complete the Click reaction. Wash the cells with PBS and then stain the nuclei with Hoechst 33342. Observe the samples using a fluorescence microscope: Detect EdU-labeled DNA with filters for Ex/Em = 501/525 nm (green fluorescence). Detect Hoechst-stained nuclei with filters for Ex/Em = 360/460 nm (blue fluorescence). Randomly select at least 3 fields of view and count the number of EdU-positive cells and Hoechst-stained nuclei. Calculate the percentage of EdU-positive cells (proliferating cells) relative to the total number of nuclei.

#### MitoTracker staining

Mitochondrial mass measured by staining with Mito-Tracker Green (Beyotime Biotechnology, C1048, Shanghai, China) was performed as described in instructions. Briefly, cells were incubated in with 50 nM Mito-tracker Green work solution (pre-warmed to 37°C) for 30 min in the dark. After staining, the Mito-Tracker Green staining solution was removed and fresh medium (pre-warmed to 37°C) was added. Apply Hoechst (Beyotime Biotechnology,326 Cat#C1029, Shanghai, China) for 10 min at 37 °C, and observe them under the fluorescence confocal microscope (IX71, Olympus Life Science, Tokyo, Japan).

The level of mitochondrial ROS was measured with MitoSOX (Thermo Fisher Scientific, Cat#M36007, Sunnyvale, CA, USA) by flow cytometry. Cells were incubated with MitoSOX 1 μM for 30 min at 37° C and 5% CO2, washed with PBS 3 times and analyzed in flow cytometer (BD FACS Calibur, USA).

#### Cell mitochondrial stress test

One day before the Seahorse XF experiment, seeding 1×10^4^ ENCCs per well in the Seahorse XF96 cell plate. On the second day, replace the medium with ENCC medium containing or lacking 2.5 μg/ml rFGL2.

For analysis of the Oxygen consumption rate (OCR) (in pmol/min), the Seahorse XF-96 metabolic extracellular flux analyzer was used (Seahorse Bioscience). Caco-2 and NCM40D were plated onto Seahorse cell plates (8 × 10^4^ cells per well) in serum-free unbuffered DMEM medium (Sigma-Aldrich). Perturbation profiling of the use of metabolic pathways was achieved by the addition of oligomycin (1 μM), Carbonyl cyanide-4-(trifluoromethoxy) phenylhydrazone (FCCP) (0.5 μM) and rotenone (1.3 μM) plus antimycin A (20 μM) (all from Sigma-Aldrich). Experiments with the Seahorse system were done with the following assay conditions: 2 min mixture; 2 min wait; and 4–5 min measurement. Metabolic parameters were then calculated as previously described.

One day before the Seahorse XF experiment, seeding 1×10^4^ ENCCs per well in the Seahorse XF96 cell plate. On the second day, replace the medium with ENCC medium containing or lacking 2.5 μg/ml rFGL2.

One day before the experiment, hydrate the sensor plate by adding 200 μL of sterile water, ensuring proper hydration, and incubate it in a 37°C, non-CO2 cell culture incubator overnight. Preheat the Seahorse XFe96 assay system at 37°C for at least 5 h. During the experiment, replace sterile water in the sensor plate with Seahorse XF calibration solution, and switch the ENCC medium in the cell plate with Seahorse XF assay medium.

The Seahorse XF DMEM Medium for mitochondrial stress testing consists of 97 mL Seahorse XF DMEM Medium (103575-100, Agilent Technologies) + 1 mL glucose (103577-100) + 1 mL pyruvate (103578-100) + 1 mL glutamine (103579-100), pH 7.4. The detection solution for glycolytic stress is Seahorse XF DMEM Medium (103575-100) + 2 mM glutamine (103579-100), pH 7.4. Place the cells in a non-CO2 cell culture incubator at 37°C for 60 min to achieve equilibrium.

To assess cellular oxygen consumption rate (OCR), the Mito Stress Test reagent kit (103015-100, Agilent Technologies) is utilized. Continuous measurements are made in the presence of glucose, and every 5 min before and after the sequential introduction of oligomycin (1.5 μM), FCCP (1 μM), and rotenone/antimycin A (0.5 μM). Experiments with the Seahorse system were done with the following assay conditions: 2 min mixture; 2 min wait; and 4–5 min measurement. Metabolic parameters were then calculated as previously described.

#### Terminal deoxynucleotidyl transferase– mediated dUTP nick-end labeling (TUNEL) staining

TUNEL assay was carried out using one-step TUNEL Apoptosis Assay Kit (Beyotime, Cat#C1089) and performed as previously describe. Briefly, ENCCs cells were cultured onto 12-well plates overnight for attachment and treated with rFGL2 for 6 h. After fixation with 4% paraformaldehyde solution at room temperature, the cells were incubated with TUNEL mixture for 1 h and stained with DAPI for 10 min in the dark subsequently. The TUNEL positive cell ratio was determined by quantifying the percentage of TUNEL labeled cells after being photographed with a fluorescence confocal microscope (IX71, Olympus Life Science, Tokyo, Japan).

#### RNA sequencing

##### Study design

Colonic tissue specimens were obtained from three pediatric patients diagnosed with Hirschsprung disease (HSCR, OMIM 142623) during definitive surgical resection. For each patient, paired samples were collected from the aganglionic (diseased) segment and the ganglionic (proximal, unaffected) segment, designated as “X” and “K,” respectively. This yielded a total of six tissue samples: X-1/K-1, X-2/K-2, and X-3/K-3.

ENCCs were isolated from the gut tissues of embryonic day 14–15 C57BL/6J mice and cultured in complete ENCC medium under defined conditions. Cellular identity was confirmed by immunofluorescence staining for P75 and nestin, with >95% of cells expressing both markers. Upon reaching 80–90% confluence, cultures were randomly allocated into two groups: (1) FGL2-treated group, in which ENCCs were stimulated with recombinant fibrinogen-like protein 2 (FGL2), and (2) control group, cultured under identical conditions without FGL2. Each group comprised three biological replicates.

#### RNA extraction library construction and sequencing

Total RNA was extracted using Trizol reagent kit (Invitrogen, Carlsbad, CA, USA) according to the manufacturer’s protocol. RNA quality was assessed on an Agilent 2100 Bioanalyzer (Agilent Technologies, Palo Alto, CA, USA) and checked using RNase-free agarose gel electrophoresis. After total RNA was extracted, eukaryotic mRNA was enriched by Oligo (dT) beads. Then the enriched mRNA was fragmented into short fragments using fragmentation buffer and reversly transcribed into cDNA by using NEBNext Ultra RNA Library Prep Kit for Illumina (NEB #7530, New England Biolabs, Ipswich, MA, USA). The purified double-stranded cDNA fragments were end repaired, A base added, and ligated to Illumina sequencing adapters. The ligation reaction was purified with the AMPure XP Beads (1.0×). And polymerase chain reaction (PCR) amplified. The resulting cDNA library of colon tissue sequencing assay was sequenced using Illumina Novaseq6000 by Beijing igeneCode Biotech Co., Ltd. (Beijing, China). The resulting cDNA library of cell sequencing assay was sequenced using Illumina Novaseq6000 by Gene *Denovo* Biotechnology Co., Ltd. (Guangzhou, China).

#### RNA-seq analysis

Total RNA extracted from human gut tissue was quantified and qualified prior to sequencing. Library preparation and RNA-seq were performed using the Illumina HiSeq platform. The paired-end sequencing was conducted with a read length of 2 × 150 bp. An average sequencing depth of approximately 6.21 Gbp per sample was obtained, ensuring sufficient coverage for robust downstream analyses.

Then sequence quality control was verified using FastQC (version 0.18.0).^33^ Subsequently, low-quality reads were filtered out based on the following criteria.

(1) Removing reads containing adapters;
(2) Removing reads containing more than 5% of unknown nucleotides(N);
(3) Removing low quality reads containing more than 20% of low quality (Q-value ≤10) bases.

The remaining high-quality reads (referred to as clean reads) were used for downstream analysis.

Clean reads were aligned to the human reference genome (GRCh38/hg38) using HISAT (v2.0.4).

Transcriptome assembly was performed for each sample using StringTie (v1.2.1). The resulting transcript assemblies from all samples were then merged using Cuffmerge (v2.2.1), followed by comparison with the reference annotation using Cuffcompare. Transcripts with class codes ‘u’, ‘i’, ‘o’, and ‘j’ were defined as novel transcripts.

Coding potential of the novel transcripts was predicted using CPC (v0.9-r2). Transcripts predicted to have coding potential were incorporated into the reference gene annotation to generate a comprehensive transcript set for expression quantification.

Clean reads were aligned to the reference transcriptome using Bowtie2 (v2.2.6), and gene and transcript expression levels were subsequently quantified using RSEM (v1.2.12).

Differential expression analysis was performed using DESeq2 (v1.10.1) in R (v4.0.5). Genes with |log2FoldChange| ≥ 1.0 and P-value <0.05 were considered differentially expressed.

Total RNA extracted from cultured cells was quantified and qualified prior to sequencing. Library preparation and RNA-seq were performed using the Illumina HiSeq platform. The paired-end sequencing was conducted with a read length of 2 × 150 bp. An average sequencing depth was approximately 6.35 Gbp per sample was achieved, ensuring sufficient coverage for downstream analyses.

Sequence quality control was performed using Fastp (v0.18.0) with the following filtering criteria.

(1) Removing reads containing adapter sequences;
(2) Removing reads with more than 10% ambiguous bases (N);
(3) Removing reads composed entirely of adenine (poly-A reads)
(4) Removing low-quality reads in which more than 50% of the bases had a Phred quality score ≤20.

To remove ribosomal RNA (rRNA) contamination, clean reads were aligned to the species-specific rRNA database using the short-read alignment tool Bowtie2 (v2.28), with no mismatches allowed. Reads that aligned to rRNA sequences were discarded, and the remaining unmapped reads were retained for downstream transcriptome analysis.

Clean reads were aligned to the mouse genome (GRCm39, Ensembl release 111) using HISAT2 (v2.1.0). Based on the alignment results, transcript reconstruction was performed using StringTie (v1.3.4), and gene expression levels for each sample were quantified using RSEM (v1.2.19).

Differential expression analysis was performed using DESeq (v1.20.0), based on read count data. Genes with |log2FoldChange| ≥ 1.5 and P-value <0.05 were considered differentially expressed.

For pathway analysis, Gene Set Enrichment Analysis (GSEA, v2.2.4) was conducted using the latest KEGG and GO databases, and FDR q-values were reported for significance assessment. And the pathways with normalized ES > 1.0 and *p*-value <0.05 were considered significantly different.

### Quantification and statistical analysis

Results were presented as mean ± SEM. The normality of the data was assessed using the Shapiro-Wilk test in SPSS (version 27.0, IBM Corporation, USA). For data with a normal distribution, statistical differences were evaluated using a one-way ANOVA or an unpaired two-tailed t-test (GraphPad Prism v.8). In cases where the data did not follow a normal distribution, an unpaired nonparametric Mann–Whitney U test was applied (GraphPad Prism v.8). Significance reported as ∗*p* < 0.05, ∗∗*p* < 0.01, ∗∗∗*p* < 0.001, ∗∗∗∗*p* < 0.0001, ns: no significance. The corresponding statistical tests and definitions of significance levels are indicated in each figure legend.

## Supplemental information

Document S1. Figures S1–S7 and Tables S1 and S2

Table S3. GSEA results based on RNA-seq data of ENCCs under FGL2 stimulation

Table S4. DEG results based on RNA-seq data of ENCCs under FGL2 stimulation
