# Supplementary material for: FGL2-induced metabolic dysregulation in enteric neural crest cells provides insight into Hirschsprung disease pathogenesis
Source: iScience. 2025 Aug 22;28(9):113423. doi: 10.1016/j.isci.2025.113423 (PMC12446381; doi:10.1016/j.isci.2025.113423)
Supplement: Document S1. Figures S1–S7 and Tables S1 and S2 [file mmc1.pdf]

iScience, Volume *28*

Supplemental information

FGL2-induced metabolic dysregulation

in enteric neural crest cells provides insight

into Hirschsprung disease pathogenesis

Jichang Han, Xiaoyang Liu, Yixuan Wang, Qiongqian Xu, Dong Sun, Xintao Zhang, Xixi He, Chuncan Ma, Xue Ren, Jian Wang, Yaru Mou, Qiangye Zhang, Dongming Wang, Weijing Mu, Peimin Hou, and Aiwu Li

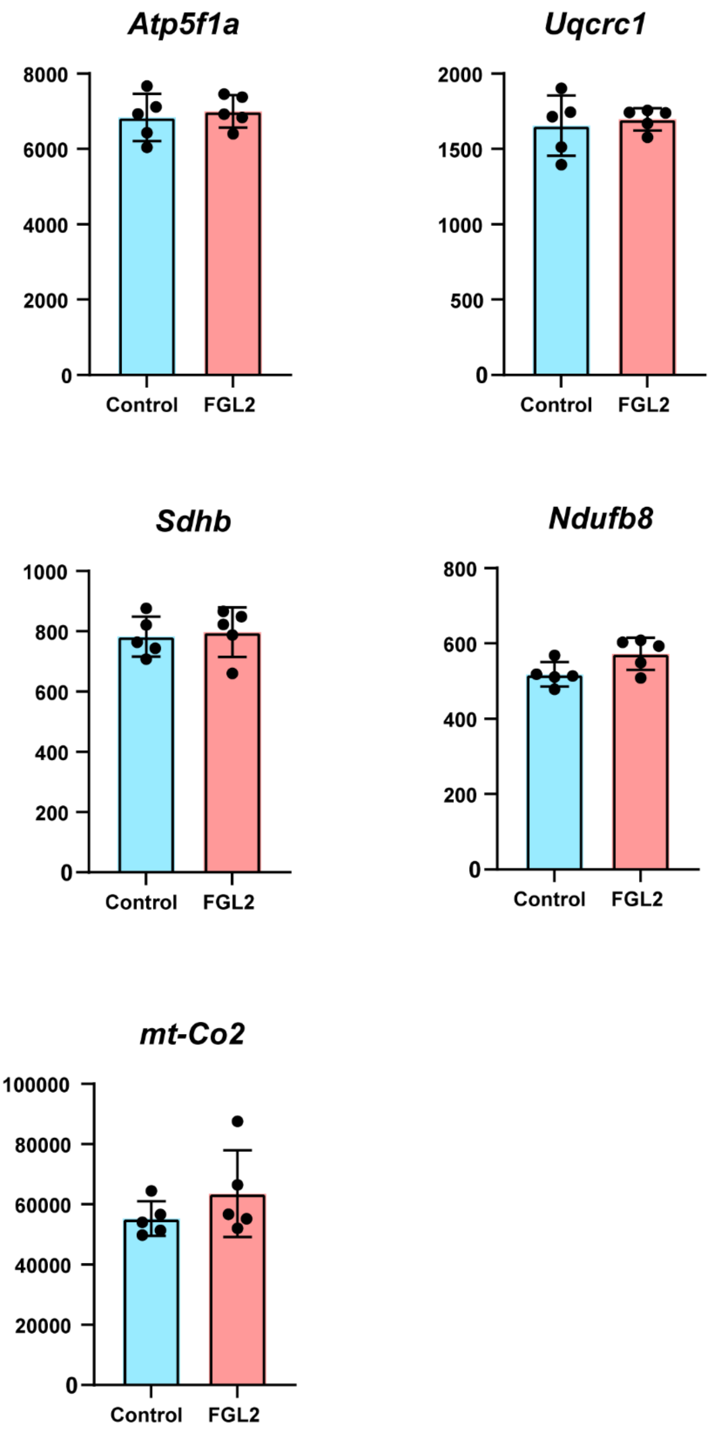

Figure S1. Normalized expression levels of OXPHOS-related genes exhibit an upward trend in the RNA-seq dataset, related to Figure 2.

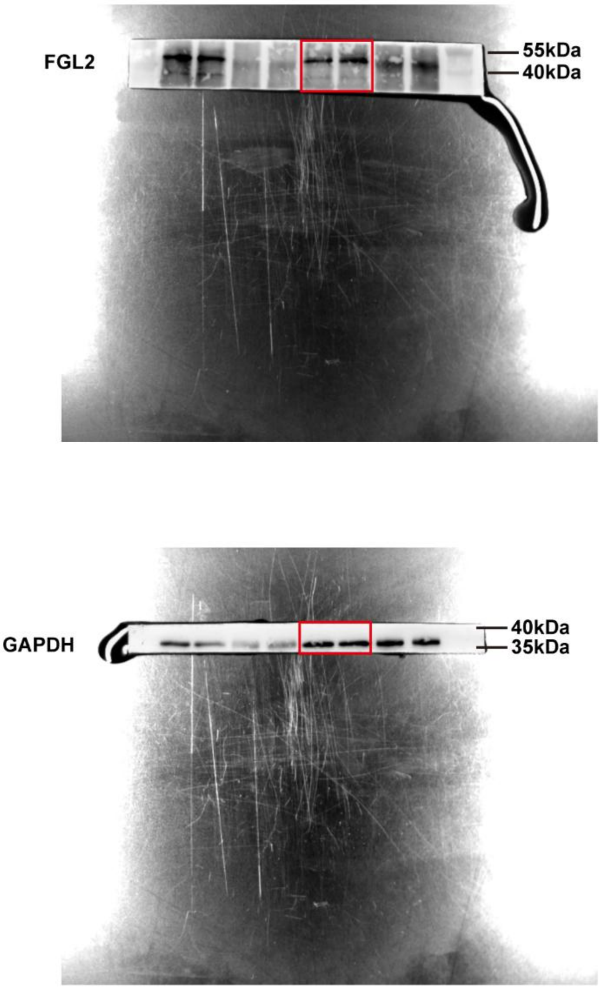

Figure S2. Original Western Blot Images, related to Figure 1.

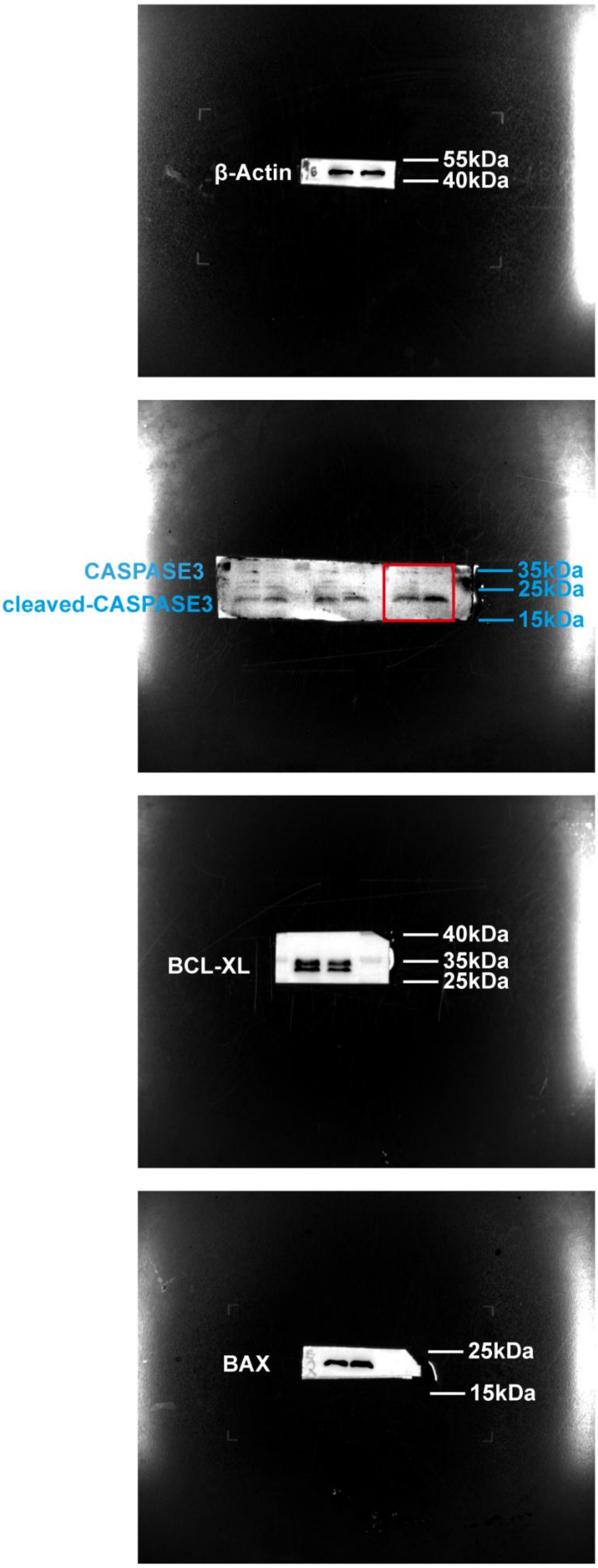

Figure S3. Original Western Blot Images, related to Figure 2.

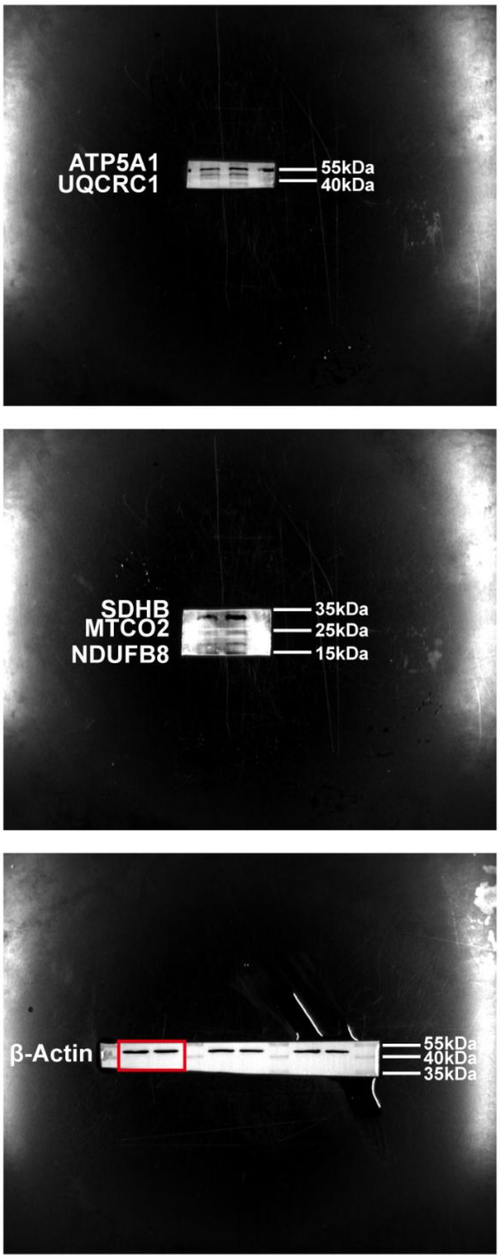

Figure S4. Original Western Blot Images, related to Figure 3.

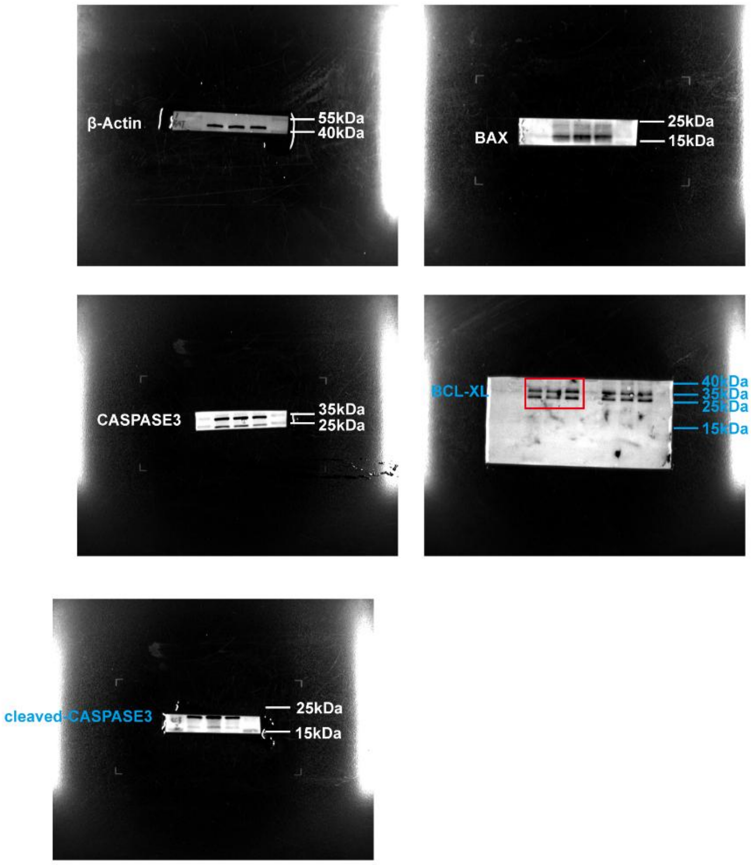

Figure S5. Original Western Blot Images, related to Figure 5.

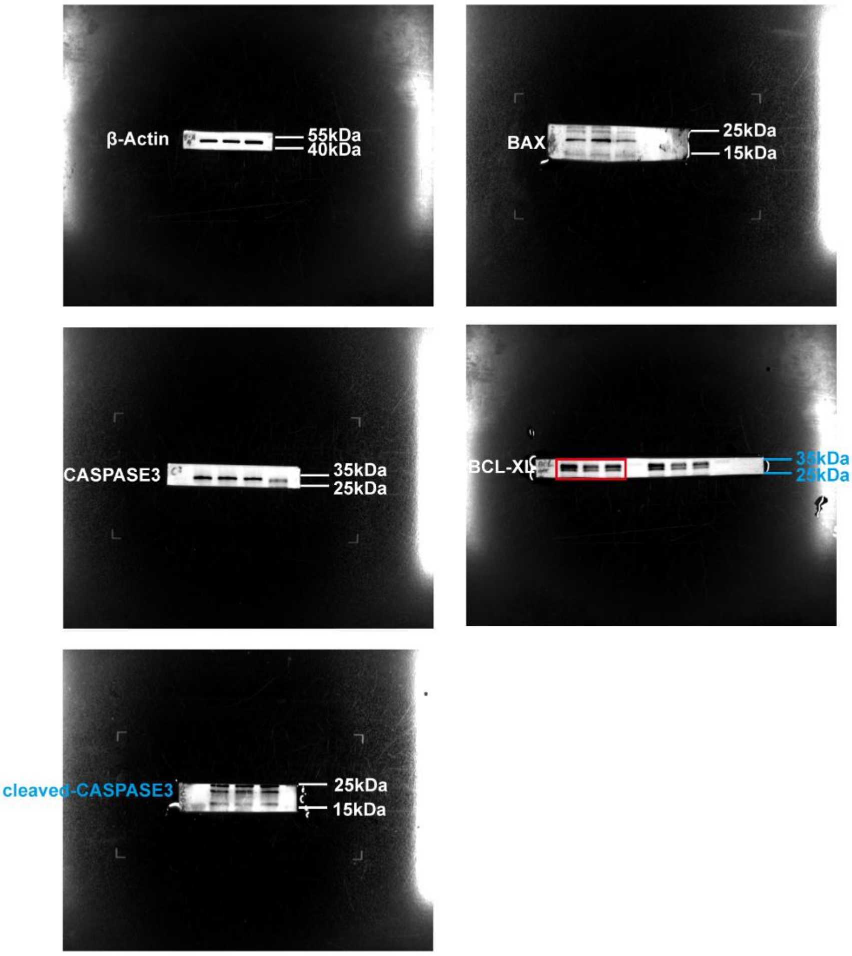

Figure S6. Original Western Blot Images, related to Figure 6.

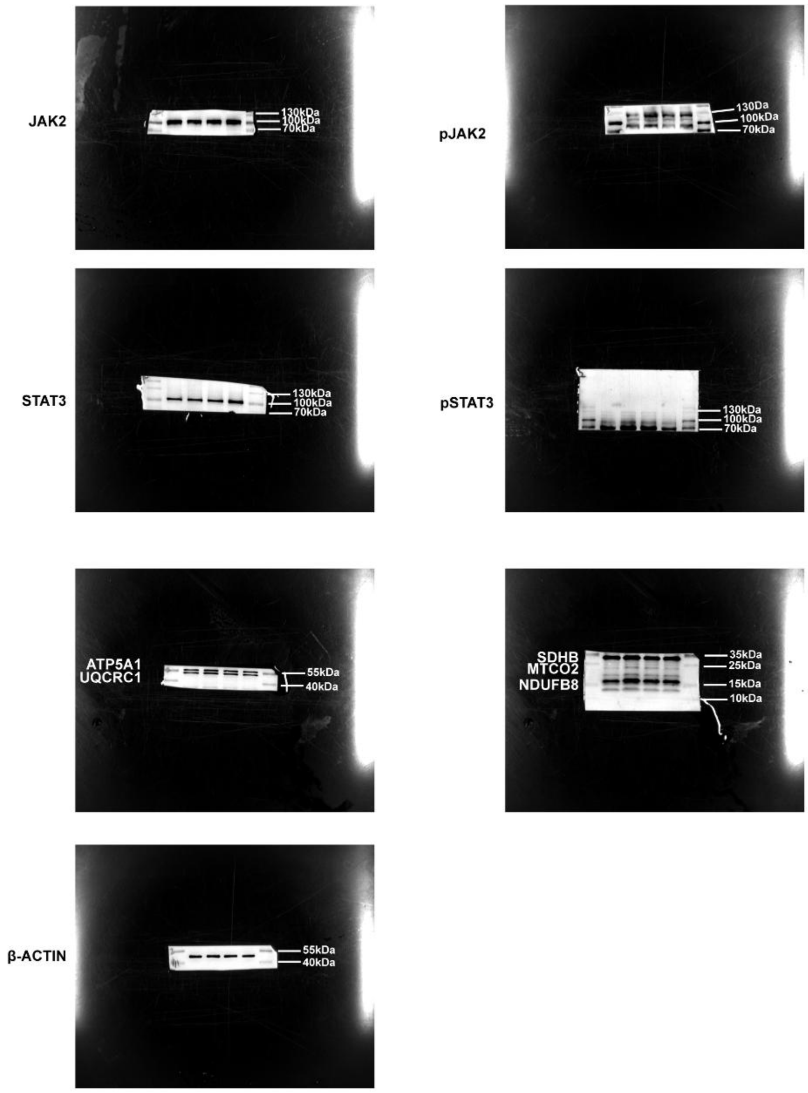

Figure S7. Original Western Blot Images, related to Figure 7.

Table S1. Clinical information of human samples used in this study, Related to STAR Methods.

Patient ID	Sex	Age at Surgery (months)	Disease	HSCR Type	Tissue Collected region (Aganglionosis)	Tissue Collected region (Ganglionosis)	Sample used for
1	F	8	HSCR	S-HSCR	Sigmoid colon	Descending colon	RNA-seq,Western blot, PCR, IHC
2	M	12	HSCR	L-HSCR	Sigmoid colon	Descending colon	RNA-seq,Western blot, PCR, IHC
3	M	3	HSCR	S-HSCR	Sigmoid colon	Descending colon	RNA-seq,Western blot, PCR, IHC
4	M	8	HSCR	L-HSCR	Sigmoid colon	Descending colon	Western blot, PCR, IHC, ELISA
5	M	9	HSCR	S-HSCR	Sigmoid colon	Descending colon	Western blot, PCR, IHC, ELISA
6	M	3	HSCR	L-HSCR	Sigmoid colon	Descending colon	Western blot, PCR, IHC, ELISA
7	M	0	HSCR	TCA	Sigmoid colon	Ascending colon	Western blot, PCR, IHC, ELISA
8	F	1	HSCR	L-HSCR	Sigmoid colon	Descending colon	Western blot, PCR, IHC, ELISA
9	F	12	HSCR	S-HSCR	Sigmoid colon	Descending colon	Western blot, PCR, IHC, ELISA
10	F	16	HSCR	S-HSCR	Sigmoid colon	Descending colon	Western blot, PCR,

							IHC
11	M	1	HSCR	S-HSCR	Sigmoid colon	Descending colon	Western blot, PCR, IHC
12	M	0	HSCR	S-HSCR	Sigmoid colon	Descending colon	Western blot, PCR, IHC
13	M	0	HSCR	L-HSCR	Sigmoid colon	Descending colon	Western blot, PCR, IHC
14	F	0	HSCR	L-HSCR	Sigmoid colon	Descending colon	Western blot, PCR, IHC
15	M	9	HSCR	S-HSCR	Sigmoid colon	Descending colon	Western blot, PCR, IHC
16	M	15	Cryptorchidism				ELISA
17	M	22	Cryptorchidism				ELISA
18	F	13	Hernia				ELISA
19	M	30	Hernia				ELISA
20	M	17	Hernia				ELISA
21	M	12	Ureteral calculus				ELISA

Table S2. Primer sequences for qRT-PCR, Related to STAR Methods.

**Gene name**	**Sequence 5'-3'**
human *GAPDH* forward	GCACCGTCAAGGCTGAGAAC
human *GAPDH* reverse	TGGTGAAGACGCCAGTGGA
human *FGL2* forward	AGCAGAGGGAAATGCGAAGA
human *FGL2* reverse	CCTGCTGAATTGCTTCGGGA
mouse *β-actin* forward	GGCTGTATTCCCCTCCATCG
mouse *β-actin* reverse	CCAGTTGGTAACAATGCCATGT

mouse *Bax* forward	ACCAGGGTGGCTGGGAAG
mouse *Bax* reverse	CCTTTCCCCTTCCCCCATTC
mouse *Bcl-xl* forward	GAGAGGCAGGCGATGAGTTT
mouse *Bcl-xl* reverse	AAAGCTCTGATACGCGGTCC
mouse *Caspase3* forward	CATGGGAGCAAGTCAGTGGA
mouse *Caspase3* reverse	TGACATTCCAGTGCTCTTATGGA
mouse *Atp5f1a* forward	CCTGAACTTGGAACCCGACA
mouse *Atp5f1a* reverse	ATAGCATTACCGAGGGCGTC
mouse *Uqcrc1* forward	ACTCGGGGCAAAAACATCCT
mouse *Uqcrc1* reverse	GCAAATGTCACGCAGCATCT
mouse *Ndufb8* forward	ATGTTGCCGGGGTCATATCC
mouse *Ndufb8* reverse	ATCGGGGTATGGCTCGTAGT
mouse *mt-Co2* forward	ACGAAATCAACAACCCCGTA
mouse *mt-Co2* reverse	TGGAAGTTCTATTGGCAGAACGA
mouse *Sdhb* forward	GACTTCACAGAGGAACGCCT
mouse *Sdhb* reverse	GCTCGCTTCTCCTTGTAGGT

Table S3. GSEA results based on RNA-seq data of ENCCs under FGL2 stimulation.

Table S4. DEG results based on RNA-seq data of ENCCs under FGL2 stimulation.
